# Effect of Defatted Dabai Pulp Extract in Urine Metabolomics of Hypercholesterolemic Rats

**DOI:** 10.3390/nu12113511

**Published:** 2020-11-14

**Authors:** Noor Atiqah Aizan Abdul Kadir, Azrina Azlan, Faridah Abas, Intan Safinar Ismail

**Affiliations:** 1 Department of Nutrition, Faculty of Medicine and Health Sciences, Universiti Putra Malaysia, UPM, Serdang 43400, Malaysia; atiqahaizan@yahoo.com; 2 Research Centre for Excellence for Nutrition and Non-communicable Disease, Faculty of Medicine and Health Sciences, Universiti Putra Malaysia, UPM, Serdang 43400, Malaysia; 3 Halal Products Research Institute, Universiti Putra Malaysia, UPM, Serdang 43400, Malaysia; 4 Department of Food Sciences, Faculty of Food Science and Technology, Universiti Putra Malaysia, UPM, Serdang 43400, Malaysia; faridah_abas@upm.edu.my; 5 Department of Chemistry, Faculty of Science, Universiti Putra Malaysia, UPM, Serdang 43400, Malaysia; safinar@upm.edu.my

**Keywords:** antioxidant, anti-inflammatory, defatted dabai pulp, hypercholesterolemia, NMR metabolomics, supercritical fluid extraction, total dietary fibre

## Abstract

A source of functional food can be utilized from a source that might otherwise be considered waste. This study investigates the hypocholesterolemic effect of defatted dabai pulp (DDP) from supercritical carbon dioxide extraction and the metabolic alterations associated with the therapeutic effects of DDP using ^1^H NMR urinary metabolomic analysis. Male-specific pathogen-free Sprague–Dawley rats were fed with a high cholesterol diet for 30 days to induce hypercholesterolemia. Later, the rats were administered with a 2% DDP treatment diet for another 30 days. Supplementation with the 2% DDP treatment diet significantly reduced the level of total cholesterol (TC), triglyceride, low-density lipoprotein (LDL), and inflammatory markers (C-reactive protein (CRP), interleukin 6 (IL6) and tumour necrosis factor-α (α-TNF)) and significantly increased the level of antioxidant profile (total antioxidant status (TAS), superoxide dismutase (SOD), glutathione peroxide (GPX), and catalase (CAT)) compared with the positive control group (PG) group (*p* < 0.05). The presence of high dietary fibre (28.73 ± 1.82 g/100 g) and phenolic compounds (syringic acid, 4-hydroxybenzoic acid and gallic acid) are potential factors contributing to the beneficial effect. Assessment of ^1^H NMR urinary metabolomics revealed that supplementation of 2% of DDP can partially recover the dysfunction in the metabolism induced by hypercholesterolemia via choline metabolism. ^1^H-NMR-based metabolomic analysis of urine from hypercholesterolemic rats in this study uncovered the therapeutic effect of DDP to combat hypercholesterolemia.

## 1. Introduction

Dyslipidemia is the leading cause of atherosclerotic cardiovascular disease (CVD) [1]. The development of premature cardiovascular disease was associated with high plasma concentrations of cholesterol, and above all, low-density lipoprotein (LDL) cholesterol [2]. In Malaysia, the latest National and Health Morbidity Survey (NHMS) 2019 reported that the prevalence of “known hypercholesterolemia” increased to 13.5% in 2019 from 9.1% in 2015. Alarmingly, CVD is the leading cause of death in Malaysia (such as stroke and coronary heart diseases) [3]. Commonly used drugs for the treatment of hypercholesterolemia and patients with a high risk of cardiovascular disease (CVD) are hydroxy-methyl-glutaryl-coenzyme-A (HMG-CoA) reductase inhibitors or statins [4]. However, many patients may not use pharmacological therapies such as statins, fibrates, bile acid resin binders, or ezetimibe in the management of dyslipidemia. The European Atherosclerosis Society stated that, in the past ten years, several observational studies had uncovered the unfavourable side effects associated with statins which include musculoskeletal disorders (statin-associated muscle symptoms), gastrointestinal disorders, and fatigue [5]. Hence, a new target for prevention and new agents for therapy with selective effect against elevated cholesterol need to be identified. In this context, the utilization of plants that offer lipid-lowering effects seems to be an excellent strategy for CVD control.

*Canarium odontophyllum* Miq. fruit, also known as “dabai”, is a native seasonal fruit that can only be found on Borneo Island, especially in the Sibu and Kapit regions of Sarawak, Malaysia. Dabai fruit is recognized as Sarawak olive or Sibu olive due to similar physical appearance, flavours, and texture with olives [6]. Defatted dabai pulp (DDP) is a waste obtained from the extraction of dabai pulp oil. DDP contains anthocyanins as the major antioxidants [7], and is high in dietary fibre [8]. Earlier, hypercholesterolemic rabbits receiving anthocyanin-rich DDP showed a cholesterol-lowering effect (reduced plasma LDL-C, and TC levels) as well as reduced atherosclerotic plaques [8]. Moreover, the extracts of DDP have been demonstrated to exhibit antioxidative properties and cardioprotective effects [9]. Supercritical carbon dioxide-extracted (SC-CO_2_) DDP is a toxic-free alternative that could offer a lipid-lowering effect and plausible to be investigated as a potential source for the formulation of nutraceuticals. The present study aimed to investigate the hypocholesterolemic effect of DDP from SC-CO_2_ extraction. Additionally, to the best of our knowledge, this is the first study to illustrate the possible metabolic pathways using ^1^H NMR-based urine metabolomics technique in response to 2% DDP treatments in the hypercholesterolemic rats.

## 2. Materials and Methods

### 2.1. Collection of Defatted Dabai Pulp

Fresh dabai fruits (226 kg) were collected from Sarikei Sarawak, Malaysia. The authoritative identification and collection of the fruits were aided by research officers of the Agriculture Research Centre (ARC), Semongok, Sarawak, Malaysia. Dabai fruits were properly stored in airtight chilled containers and dispatched to the Faculty of Medicine and Health Sciences, Universiti Putra Malaysia. Dabai fruits without physical damage were examined and selected. The seed was removed and dabai pulps were sent to Phytes Biotek Sdn Bhd for freeze-drying by using an industrial scale freeze dryer (VirTis BM 5000, Warminster, PA, USA).

Next, freeze-dried dabai pulp was ground together to produce no less than 0.2 mm powder. The ground powder (62.46 kg) was subjected to a large-scale supercritical carbon dioxide (SC-CO_2_) extraction at Supercritical Fluid Centre (SFC) Universiti Putra Malaysia. The SC-CO_2_ extraction was performed at 40 °C and with an extraction pressure of 40 MPa. The extraction condition was performed based on the method described in our previous study [10]. DDP is a waste that resulted in following large-scale supercritical carbon dioxide (SC-CO_2_) extraction. DDP (40 kg) was collected and stored at 4 °C until further analysis.

### 2.2. Nutritional Quality of Defatted Dabai Pulp (DDP)

DDP was analysed for its total dietary fibre and individual phenols. Total dietary fibre of DDP was analysed according to AOAC 16th Edition, 991.43–Enzymatic–Gravimetric Method—MES-TRIS Buffer [11].

Individual phenols were analyzed by high-performance liquid chromatography (HPLC) [12]. DDP (0.5 g) was placed into a polyethylene centrifuge tube (13 mL) containing 5 mL of 62.25% methanol. After vigorous shaking for 1 min by using a vortex, the sample was directly sonicated using POWERSONIC 405 (Hwashin Technology Seoul, South Korea) ultrasonic for 15 min. Later, the sample was centrifuged at 5000 rpm for 25 min. Then, the methanolic phase was filtered through a 0.45 µm pore size and 17-mm diameter nylon filter [7]. The extract was collected into a 5 mL amber small glass vial (JUSTO, Shanghai, China).

An Agilent 1100 series (Agilent Technologies, Germany) chromatograph equipped with a diode-array detector (DAD) was used. A gradient of solvent A (Water–Methanol–Acetic acid 93:5:2 *v/v/v*) and solvent B (Methanol–Acetic acid 98:2 *v/v*) was applied to a reversed-phase Lichrospher C-18 column (250 × 4 mm i.d., 5 μm) (Merck KGaA, Darmstadt, Germany) as follows: 0 min, 100% A:0%B; 30 min, 60% A:40%B; 32 min, 100% A:0%B. The flow rate was 1 mL min^−1^ and the column temperature was set at 30 °C. The volume injected was 20 μL. The selection of phenolic compounds (gallic acid, 4-hydroxybenzoic acid, and syringic acid) was chosen based on the phenolic acids that are found in dabai fruits [7,12,13]. Quantification of phenolic compounds was carried out using the area values measurements at 280 nm. The identification of chromatographic peaks was carried out by comparing their retention times and spectra with those of standards. Quantitative assays were achieved using external calibration curves for all standard phenols.

### 2.3. Antioxidant Profile in Treatment Diets

The preparation of the treatment diet was described in Section 2.4. The treatment diets were freeze-dried and ground into powder. The powdered treatment diet (0.1 g) was placed into a polyethylene centrifuge tube (13 mL) containing 1 mL of 80% methanol. The mixture was sonicated by using POWERSONIC 405 (Hwashin Technology Seoul, South Korea) ultrasonic for 15 min, vortexed each for 30 s, and centrifuged for 5 min at 5000 rpm. The supernatants were collected into a 5 mL flask [14]. The extract was used for the determination of antioxidant activity, total phenolic content, and total flavonoid content.

The antioxidant activity in the treatment diet was analysed by ferric ion reducing antioxidant power (FRAP)[15]. The treatment diet extract (0.1 mL) was transferred in the volumetric flask. Later, 1 mL of FRAP reagent was placed into the volumetric flask. The sample was vortexed for a few seconds to completely mix the sample and reagents. The mixture was incubated at 37 ℃. After 4 min, the mixture was measured at 593 nm by using a spectrophotometer. The same procedure was used for FeSO_4_.7H_2_O (0.1 mM—1.0 mM) for a calibration curve and the result was expressed as Mm ferrous equivalent per g/extract (mM Fe/g extract).

The total phenolic content of the methanol extracts of the treatment diet was determined by employing the method involving Folin–Ciocalteu reagent and gallic acid as standard [15]. Methanolic extract of treatment diet (0.2 mL) was added to a test tube and mixed with 1.5 mL Folin–Ciocalteu reagent. After 5 min of incubation, 1.5 mL Na_2_CO_3_ (20%) solution was added. After 2 h, the absorbance was measured at 765 nm by using a spectrophotometer. The same procedure was used for gallic acid (0.05 mg–1.5 mg/mL) for a calibration curve and the result was expressed as mg gallic acid equivalent (GAE) per g extract (mg GAE/g extract).

Meanwhile, the total flavonoid content of methanol extracts of the treatment diet was estimated spectrophotometrically by the aluminium chloride method [16]. Exactly 1 mL of 2% AlCl_3_ in methanol was mixed with the same volume of the treatment diet methanolic extracts. Absorption readings at 430 nm were taken after 15 min by using a spectrophotometer. The same procedure was used for quercetin (0.05 mg–1.5 mg/mL) for a calibration curve and the result was expressed as mg quercetin equivalent (QE) per g extract (mg QE/g extract).

### 2.4. Animal Experiments

Male-specific pathogen-free (SPF) Sprague–Dawley rats at the age of 4 weeks, weighing between 100 to 150 g were purchased from Nomura Siam International Co., Ltd., Thailand. The rats were individually housed under individual Ventilated Cages (IVC) in the Comparative Medicine and Technology Unit (COMeT) Universiti Putra Malaysia with controlled conditions (21–23 °C, 50–60% relative humidity and controlled 12 h light–dark cycle) throughout the experiments. All rats were acclimatized for two weeks. Foods and water were provided *ad libitum.* All experimental protocols and ethical aspects were conducted following the proper use and care of laboratory animals, as approved by the IACUC, Universiti Putra Malaysia (IACUC R045/2015).

Following acclimatization, the animals were randomly divided into normal rats (NG; *n* = 6), which received a cholesterol-free diet (ND) whereas, the remaining rats (*n* = 12) received a high cholesterol diet (HC) containing 1% cholesterol for 30 days of hypercholesterolemia induction. The cholesterol-free diet was prepared from a mixture of corn starch, sucrose, casein, cellulose, mineral mixture, vitamin mixture, DL-methionine, choline, corn oil, and ghee. The experimental diet was prepared weekly, and all ingredients were mixed thoroughly, spread in trays, cut into smaller pieces, and baked in an oven (Binder ED23, Tuttlingen, Germany) at 50–60 °C for 24 h [17]. Similarly, with the addition of 1% cholesterol, high cholesterol diets were baked using the above process. During the animal study, all rats were given around 25 g of the respective diets by measuring both the allocated feed and leftover daily. All diets were stored at 4 °C, and fresh pallets were provided daily to the rats. After 30 days of the induction period, all experimental rats fasted overnight. Then, the rats were intraperitoneally anaesthetized with ketamine (50 mg/kg body weight) and xylazine (10 mg/kg body weight) by a veterinarian. Blood (1 mL) was collected via cardiac puncture for hypercholesterolemia screening. Rats with total serum cholesterol and LDL-C significantly higher than NG rats were considered as hypercholesterolemic rats [18].

After confirming the establishment of the hypercholesterolemia model, the rats which were fed with high cholesterol diet were further randomized into hypercholesterolemic positive control group (PG) (*n* = 6) and hypercholesterolemic rats treated with 2% DDP group (DG) (*n* = 6). Each of the groups was provided with their respective treatment diets for another 30 days. NG group received standard diet without added cholesterol while the PG group was continually on the high cholesterol diet (HC). Meanwhile, the DG group was received a formulated 2% DDP treatment diet, in which 2% of DDP was incorporated into the high cholesterol diet (HC + 2% DDP). DDP treatment diet was prepared equivalently as a high cholesterol diet (Table 1).

At the end of the treatment period (day 60), the rats were independently housed in metabolic cages and fasted for 16 hr. Urine samples were collected and stored in urine containers containing 0.1% sodium azide solution and put in storage at −20 °C until use to prevent microbial growth. Then, the rats were intraperitoneally anaesthetized with ketamine (50 mg/kg body weight) and xylazine (10 mg/kg body weight) by a veterinarian. Blood (3 mL) was collected via cardiac puncture. The collected blood was centrifuged for 10 min at 3500 rpm at room temperature, and the serum was collected and kept at −80 °C until further use. Lastly, rats were euthanized by exsanguination via a cardiac puncture through the abdominal aorta of the heart.

### 2.5. Blood Analysis

Lipid profiles were measured by using Dimension^®^ Xpand^®^ Plus (Siemens Healthcare Diagnostics, Newark, DE, USA). The biochemical tests were performed according to the manufacturer instructions for each parameter; Cholesterol (Siemens Healthcare, DF27), TG (Siemens Healthcare, DF69A), LDL (Siemens Healthcare, DF131), and HDL (Siemens Healthcare, DF48B). AST and ALT were tested by using BioLis 24i Premium Chemistry Analyzer (BioRex Mannheim Malaysia Sdn. Bhd). The assay was performed according to the manufacturer instructions for each parameter; AST (aspartate aminotransferase EC.2.6.11 according to the International Federation of Clinical Chemistry and Laboratory Medicine [IFCC]) and ALT (alanine aminotransferase EC.2.6.11 according to IFCC). All procedures were conducted attentively and precisely according to the manufacturer’s instructions.

### 2.6. Oxidative Stress Markers Analysis

The oxidative stress markers assessed in this study were total antioxidant status (TAOS), superoxide dismutase (SOD), glutathione peroxide (GPX), and catalase (CAT). Total antioxidant status was measured by using rat TAOS ELISA kit (Hangzhou SunLong Biotech Co., Ltd., China). Superoxide dismutase was analyzed by using a rat SOD ELISA kit (Elabscience Biotechnology Inc., Wuhan, China). Meanwhile, glutathione peroxide and catalase were measured by using rat GPx assay kit and rat CAT assay kit respectively (Elabscience Biotechnology Inc., Wuhan, China). All procedures were conducted attentively and precisely according to the manufacturer’s instructions.

### 2.7. Inflammatory Markers Analysis

The inflammatory markers assessed in this study were C-reactive protein (CRP), interleukin 6 (IL-6), and tumour necrosis factor-α (α-TNF). C-reactive protein level was measured by using a rat CRP ELISA kit (Elabscience Biotechnology Inc. Wuhan, China). Whereas interleukin 6 and tumour necrosis factor-α were quantified by using rat IL-6 ELISA kit and rat α- TNF ELISA kit respectively (eBioscience, San Diego, CA, USA). All procedures were conducted attentively and precisely according to the manufacturer’s instructions.

### 2.8. ^1^H-NMR Urinary Metabolomics Analysis

The thawed urine samples were centrifuged at 3000 g for 10 min at room temperature to obtain the supernatant layer. Exactly 400 μL of the supernatant layer was pipetted into an Eppendorf tube containing 200 μL of phosphate buffer solution (0.308 g of KH_2_PO_4_ in 25 mL D_2_O, pH 7.4, containing 0.1% TSP). The Eppendorf tube was vortexed for 1 min before transferring the content into a 5-mm NMR tube (Norell, Morganton, USA) and subjected to ^1^H NMR analysis. The ^1^H NMR analysis was performed on NMR spectroscopy (Varian INOVA 500 MHz, Palo Alto, USA) at 26 °C. In order to suppress the water signal, a one-dimensional pre-saturation sequence (PRESAT) with 64 scans was employed. The total acquisition time of each ^1^H NMR was 3.53 min. All the ^1^H NMR spectra were phased, baseline corrected by using Chenomx NMR suite software version 8.3 (Chenomx Inc., Edmonton, Canada) and aligned to the TSP (internal standard) at 0 ppm. Urine sample region from 0–10.0 ppm was used with a total of 235 integrated regions per spectrum. The spectra regions at 4.60–4.95 ppm (water) and spectra regions at 5.55–5.95 ppm (urea) were eliminated, and the remaining spectral regions were divided into 0.04 ppm bins (size of binned width). The resulted binned data were converted into Microsoft Excel format.

Later, the Excel data were then imported and analyzed using SIMCA-P software Version 12.0 (Umetric, Umea, Sweden) for multivariate data analysis. Principle Component Analysis (PCA) was performed to visualize and discriminated the groups according to their metabolites, thus giving the general idea within the dataset. Later, Partial Least Squares-Discriminant Analysis (PLS-DA) was generated to discriminate and to visualize the metabolites responsible for the groups. The validation of the PLS-DA model was assessed using R^2^ (goodness of fit parameter) and Q^2^ (goodness of prediction parameter) values, whereby the model was considered as effective and reliable when these values were greater than 0.5. Further, to ensure the robustness of the model, a 100-cycle permutation test was performed. A model was considered robust when all the permute R^2^ values on the left were lower than the original point on the right, and the Q^2^ regression line had a negative intercept. The robustness was considered excellent if the R^2^Y- and Q^2^X-intercepts were less than 0.5 and 0.05, respectively [19]. The misclassification probability test also confirmed the validity of the model.

In the PLS-DA model, different groups were separated into different classes and projected into a PLS-DA score plot. The metabolites that influence the separation were identified from the loading scatter plot. Then, only the metabolites in the PLS-DA model with variable importance in the projection (VIP) of more than 0.5 were selected as putative metabolites for relative quantification [20]. The pathway analysis was generated using Metaboanalyst 4.0 software (https://www.metaboanalyst.ca/). The overrepresentation and pathway topology tests of the pathway analysis were evaluated using hypergeometric and relative-betweenness centrality, respectively [21]. The pathway library was based on the Rattus norvegicus (rat) model as the experimental subjects were Sprague–Dawley rats. The potential metabolic pathways were selected according to the impact values, which measure the metabolites’ importance in the network.

### 2.9. Statistical Analysis

Data were expressed as mean ± standard deviation (*n* = 6). Data were analyzed by using one-way ANOVA using SPSS for windows version 21. Duncan’s Multiple Range Test was used to test whether there were significant differences between the experimental groups. Values were considered statistically significant when *p* < 0.05.

## 3. Results

### 3.1. Nutritional Quality of DDP and DDP Treatment Diet

DDP is rich in dietary fibre which is postulated to be an important factor in the cholesterol-lowering effect seen in the animal model [8]. In this study, the total dietary fibre (TDF) of DDP is 28.73 ± 1.82 g/100 g. Meanwhile, as for the quantification of individual phenol in DDP (Figure S1), quantitative assays, using external calibration curves for all standard phenols, achieved; gallic acid: y = 22.633x − 2.0838 (R = 0.999), 4-hydroxybenzoic acid: y = 27.882x + 98.691 (R = 0.999), and syringic acid: y = 42.174x − 6.991 (R = 1). Syringic acid (89.87 ± 15.18 µg/mL) was found the highest in DDP, followed by 4-hydroxybenzoic acid (61.46 ± 0.04 µg/mL) and gallic acid (8.73 ± 0.13 µg/mL) (Table 2).

As expected, incorporation of 2% DDP in the treatment diet resulted in significantly higher antioxidant activity analyzed by ferric ion reducing antioxidant power (FRAP), total phenolic content (TPC), and total flavonoid content (TFC) as compared to normal diet and high cholesterol diet (*p* < 0.05) (Table 3).

### 3.2. Efficacy of 2% DDP Treatment Diet in Hypercholesterolemic Rats

As expected, rats in the PG group who received a high cholesterol diet demonstrated significant elevation of total cholesterol (TC), and LDL, in comparison with the NG group (*p* < 0.05). These results suggest that hypercholesterolemia was established in the rats using a high cholesterol diet. The hypercholesterolemic condition in the PG group caused significantly lower antioxidant status (TAS) and antioxidant enzymes (SOD, GPx, and CAT) than NG group (*p* < 0.05) thus proving the link between hypercholesterolemia and oxidative stress [22]. Further, the inflammatory markers (CRP, IL-6, and α-TNF) were significantly elevated in the serum of the PG group when compared with the NG group (*p* < 0.05) confirming the association of hypercholesterolemia with inflammation [23]. Furthermore, the PG group showed a significant elevation of aspartate transaminase (AST) and alanine transaminase (ALT) in comparison with the NG group (*p* < 0.05). Therefore, in this study, hypercholesterolemia is associated with toxicity due to elevated liver enzymes in the serum [24,25].

Table 4 shows the beneficial effects of the DDP treatment diet on hypercholesterolemic rats. Supplementation with the DDP treatment diet in the DG group for 30 days significantly reduced the TC, triglyceride, LDL, and inflammatory markers levels (CRP, IL-6, and α-TNF) when compared with the PG group (*p* < 0.05). Moreover, the level of the antioxidant status (TAS) and antioxidant enzymes (SOD, GPx, and CAT) was significantly increased in the DG group in comparison with the PG group (*p* < 0.05). These results suggest that DDP exerts a hypocholesterolemia effect and improves inflammation and oxidative stress. Furthermore, the DG group showed no significant variation of AST and ALT as compared to the NG group (*p* > 0.05). The results indicated that SC-CO_2_ DDP could, therefore, be considered as having no toxicological significance.

### 3.3. H-NMR-Based Metabolomics Analysis of Urine from Hypercholesterolemic Rats Treated with 2% DDP

**Figure 1:**
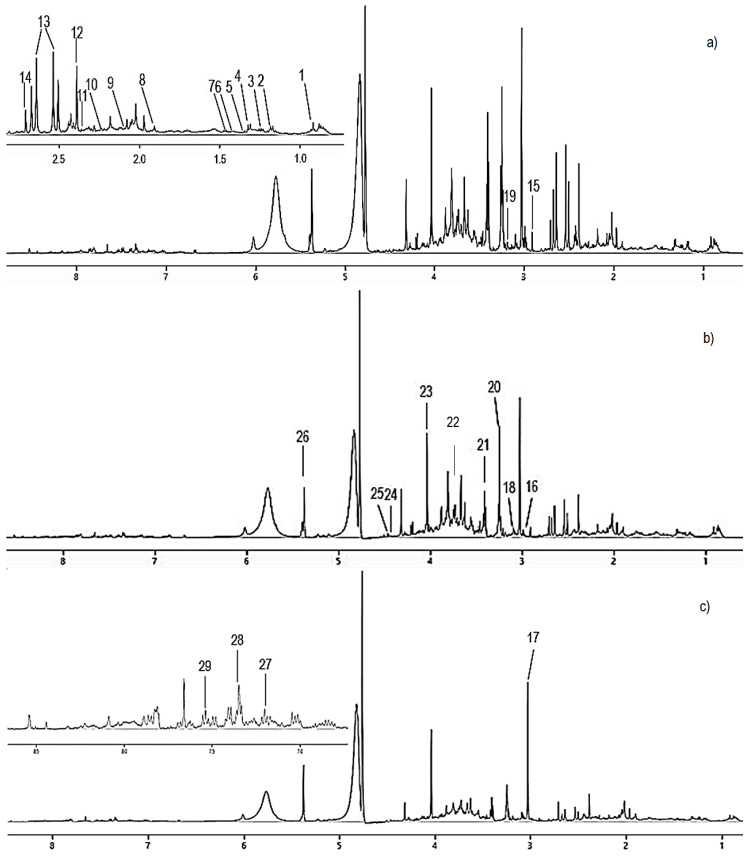
The representative of ^1^H NMR spectra of urine samples of normal group (**a**), hypercholesterolemic positive control group (**b**), and hypercholesterolemic rats treated with 2% DDP group (**c**). 1: Leucine, 2: 3-hydroxybutyrate, 3: Methylmalonate, 4: Threonine, 5: Lactate, 6: Lysine, 7: Alanine, 8: Acetate, 9: Acetone, 10: Acetoacetate, 11: Pyruvate, 12: Succinate, 13: Citrate, 14: Dimethylamine, 15: N,N-Dimethylglycine, 16: 2-oxoglutarate, 17: Creatine, 18: cis-Aconitate, 19: Choline, 20: Trimethylamine N-oxide, 21: Taurine, 22: Glucose, 23: Creatinine, 24: Trigonelline, 25: 1-Methylnicotinamide, 26: Allantoin, 27: 3-Indoxylsulfate, 28: N-Phenylacetylglycine, 29: Hippurate.

The assignment of ^1^H-NMR spectra of urine obtained from NG, PG, and DG groups is shown in Figure 1. The endogenous metabolites were assigned according to Chenomx NMR suite 6.1 (Chenomx Inc., Edmonton, AB, Canada). The metabolites should have the same shape splitting or coupling constant. Within the chemical shift range, further identification and confirmation also was made via chemical shift and coupling constant from the Human Metabolome Database (HMDB) and published assignments. Generally, the urine sample contained metabolites such as intermediate from tricarboxylate cycle (TCA) (succinate, 2-oxoglutarate, and citrate), amino acids (leucine, alanine, lysine N, N-dimethylglycine, threonine), ketone bodies (acetoacetate, 3-hydroxybutyrate [3-HB], and acetone), organic acids (taurine, hippurate, acetate, lactate, pyruvate, cis-aconitate, methylmalonate, dimethylamine), and others (allantoin, creatinine, trigonelline, choline, glucose, 1-methylnicotinamide, 3-Indoxylsulfate, creatine, N-phenylacetylglycine and trimethylamine N-oxide [TMAO]) (Table S1).

**Figure 2:**
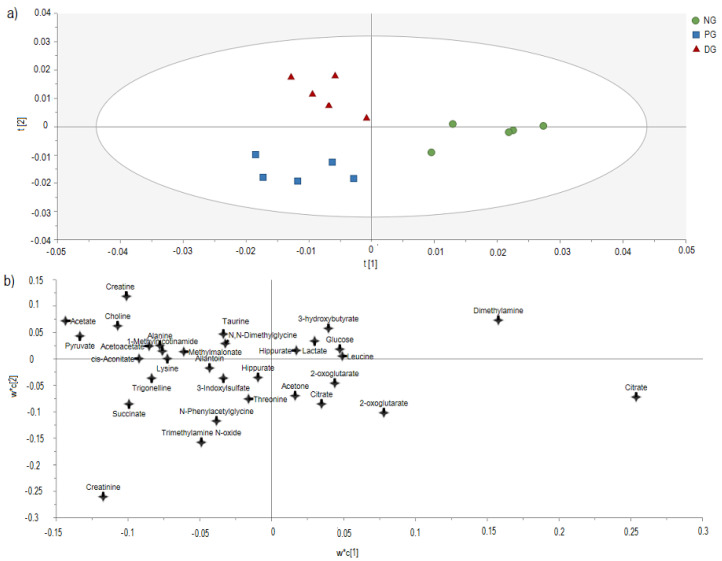
PLS-DA model. (**a**) Score plot (**b**) loading scatter plot obtained using ^1^H-NMR spectra of urine samples in normal rats group (NG), hypercholesterolemic positive control group (PG) and hypercholesterolemic rats treated with 2% DDP group (DG) after 30 days of the treatment period (R^2^Y = 0.973, Q^2^ = 0.834).

To understand the metabolite alteration between NG, PG, and DG groups, the dataset generated from the ^1^H NMR spectra was subjected to multivariate data analysis using the PLS-DA model. The generated PLS-DA score model (Figure 2a) displayed excellent goodness of fit (R^2^Y = 0.973) and predictability (Q^2^ = 0.834) values. For validity, this model possesses excellent goodness of fit (R^2^Y (cum) > 0.8) and good predictability ability (Q^2^ (cum) > 0.7). The permutation test revealed that the model demonstrated moderate model validity with R^2^Y intercept of <0.9 (0.821) and Q^2^-intercepts of <0.5 (−0.422). All the permute R^2^ values on the left were lower than the original point on the right, and the Q^2^ regression line had a negative intercept. Hence, the model was considered robust. Moreover, the misclassification table showed that the model had Fischer’s probability <0.01 (7.9 × 10^−6^), which indicates that the model was able to classify the individual subject correctly (Figure S2).

As shown in Figure 2a, all groups were separated, in which the NG group was separated from the PG group and DG group with t [1]. The PG group was clustered away from those of the NG group, indicating that hypercholesterolemia was successfully induced. The separation between PG and DG also can be observed from t [2], where DG rats are located on the positive side while PG rats are located on the negative side. The DG group also shifted away from the NG group, which indicates that the recovery had not fully returned to the normal state. The PLS-DA loading scatter plot (Figure 2b) is complementary to the PLS-DA score plot, and it describes the metabolites that influence the separation between the groups. The change of supplementation of 2% DDP can be seen in metabolites such as choline, acetate, pyruvate, methylmalonate, acetoacetate, alanine, creatine, 1-methylnicotinamide, taurine, and N, N-Dimethylglycine.

From all metabolites that had been previously identified, only metabolites with VIP values larger than 0.5 were considered as potential biomarkers. A total of nine potential biomarkers were identified with citrate having the highest VIP value (>3) followed by acetate, pyruvate, choline, cis-aconitate, acetoacetate, alanine, lysine, and methylmalonate (MMA). Prolonged intake of high cholesterol diet resulted in a significant increase of four metabolites (acetate, pyruvate, choline, and alanine) in the PG group when compared with the NG group (*p* < 0.05), whereas citrate was significantly decreased in the PG group when compared with the NG group (*p* < 0.05). Based on the variation of the metabolites mentioned above, feeding rats with high cholesterol diet (60 days) resulted in disturbance of carbohydrate metabolism, lipid metabolism, and amino acid metabolism. Meanwhile, the therapeutic effect of supplementation with 2% of DDP resulted in a significant elevation of choline when compared with PG rats (Table 5) (*p* < 0.05). Unfortunately, there was no significant difference in cis-aconitate, acetoacetate, lysine and MMA among groups.

**Figure 3:**
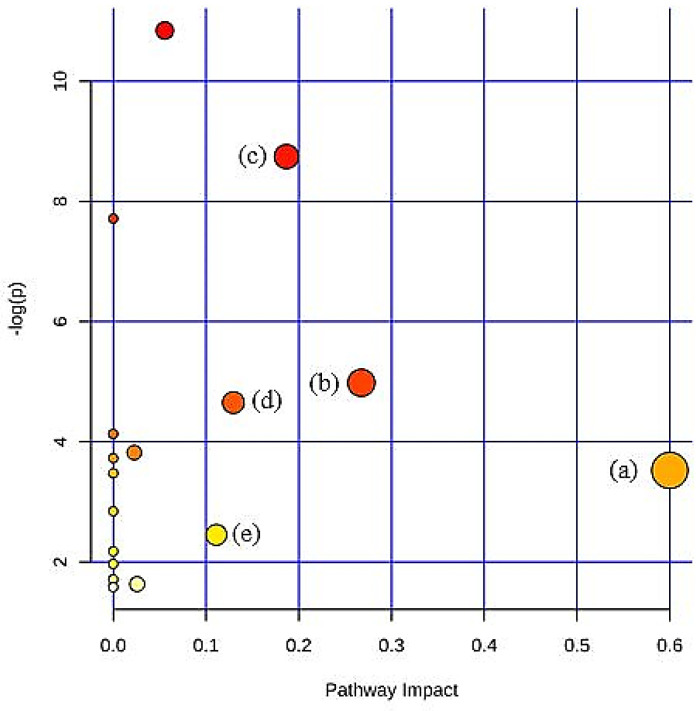
Pathways analysis (a) Synthesis and degradation of ketone bodies; (b) Pyruvate metabolism; (c) Citrate cycle (TCA cycle); (d) Glycolysis/Gluconeogenesis; (e) Butanoate metabolism.

**Figure 4:**
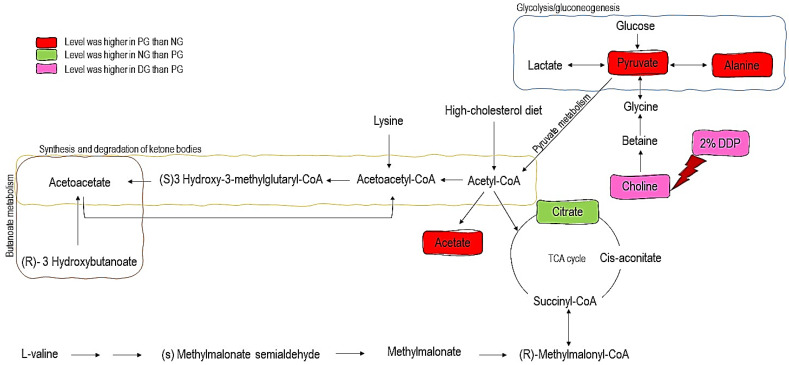
Potential metabolic pathways disturbed in hypercholesterolemic rats and alteration by 2% DDP supplementation. PG: Hypercholesterolemic positive control group; NG: Normal rats group; DG: Hypercholesterolemic rats treated with 2% DDP.

To reveal the metabolic pathways associated with the potential biomarkers identified, a pathway analysis tool (metaboanalyst software) was applied to elucidate the possible metabolic pathways in response to 2% DDP treatments in hypercholesterolemic rats. Metabolic pathways with impact values of more than 0.1 were selected as the potential pathway [21] (Figure 3). In Table 6, five metabolic pathways with impact values more than 0.1 were the pathway related to lipid metabolism (synthesis and degradation of ketone bodies), pathway related to energy metabolism (TCA cycle, pyruvate metabolism, and glycolysis/gluconeogenesis), and others (butanoate metabolism). It turned out that supplementation of 2% of DDP partially recovered the dysfunction in the metabolism induced by hypercholesterolemia via lipid metabolism. The interrelationship between the potential biomarkers and its pathways is shown in Figure 4.

## 4. Discussion

Fruit and vegetable wastes are often discarded after extraction. Ironically, the waste still contains a large number of bioactive compounds, and attempts have been made broadly to utilized fruits and vegetable waste therapeutically [26]. DDP is a waste product after the oil extraction from dabai pulp using supercritical fluid extraction. In this study, the total dietary fibre (TDF) of DDP is 28.73 ± 1.82 g/100 g. The total dietary fibre in DDP is higher than total dietary fibre in rice (dry) (1.3 g/100 g), oats (10.3 g/100 g), wheat (whole grain) (12.6 g/100 g), corn (13.4 g/100 g), soy (15.0 g/100 g), and flaxseed (22.33 g/100 g) [27,28]. Meanwhile, syringic acid (SA) was shown to be the major compound in DDP based on its high quantity (89.87 ± 15.18 µg/mL) when compared to that of 4-hydroxybenzoic acid (4-HBA) (61.46 ± 0.04 µg/mL) and gallic acid (GA) (8.73 ± 0.13 µg/mL). Finding a new affordable source of fibre that can be developed as a new source for high dietary fibre food in the food industry has become a goal for food companies to improve their product in the market and to attract customers.

The source of fibre can be utilized from the source that might otherwise be considered waste [29]. Adding fibre to foods can influence the consistency, texture, rheological behaviour, and sensory characteristics of the end products [30]. Breakfast cereals and bakery products are the most available foods that are enriched with fibre [31]. Sharif et al. [32] suggested that replacing wheat flour with defatted rice bran could be utilized without adversely affecting the physical and sensory characteristics of the cookies. Additionally, the incorporation of defatted rice bran significantly improved the dietary fibre, mineral and protein content of the cookies and cost production was also reduced as the incorporation of defatted rice bran was increased. A previous study by Nassar [33] demonstrated that an orange peel and pulp had a high amount of dietary fibre (74.87 g/100 g and 70.64 g/100 g, respectively). The fibre can be incorporated as an ingredient in making biscuits, as orange peel and pulp are suitable sources of dietary ingredients associated with bioactive compounds such as flavonoids and carotenoids. Nowadays, food containing phenolic compounds has become the main interest due to their health benefit on humans [34]. Additionally, evidence is emerging that the combination of phytochemicals is more effective in protecting against risk factors of CVD than a single phenolic compound [35,36]. This study showed that alongside with dietary fibre, bioactive compounds (SA, 4-HBA, and GA) are highly present in DDP. Hence, DDP could be a valuable and novel source of dietary fibre with associated bioactive compounds that can be utilized in a large variety of food products such as making biscuits.

Further, we demonstrated that 2% DDP ameliorate hypercholesterolemia, oxidative stress and inflammation. Supporting evidence is available that DDP has a preventive effect in hypercholesterolemic rabbits in the previous study by Shakirin et al. [8]. Further, the potent antioxidant properties in DDP that responsible for the marked increments in antioxidant enzymes were also observed in the previous study by Azlan et al. [37]. These efficiencies may be due to TDF and bioactive compounds (SA, 4-HBA, and GA) in DDP that attenuate the imbalance in the hypercholesterolemia condition.

To further explore the variations of urine metabolites in the case of hypercholesterolemia and the therapeutic effect of 2% DDP, an NMR-based metabolomics approach combined with multivariate analysis was performed to identify the potential biomarker, based on which corresponding metabolic pathways were proposed (Figure 4). Pyruvate, an essential intermediate product of glycolysis and gluconeogenesis, was frequently associated with glucometabolic. Commonly, a decreased level of pyruvate indicates an inhibited glycolysis and activated gluconeogenesis. Pyruvate can be used to produce acetyl-CoA by pyruvate dehydrogenase complex, which enters the TCA cycle and playing a pivotal role in glucose aerobic oxidation and energy production. Pyruvate can also be converted to alanine via alanine aminotransferase (ALT) as well as to lactate via lactate dehydrogenase (LDH). In the case of hyperlipidemia, a large amount of acetyl-CoA was generated, which exerts negative feedback on the activity of pyruvate dehydrogenase complex, thus inhibiting its ability to consume pyruvate, resulted in two other outputs, (alanine and lactate) [38,39]. Alanine is a glucogenic amino acid. An elevated level of alanine may stem from the transformation of acetyl-CoA, while gluconeogenesis from amino acid is suppressed [40]. Perturbation in energy metabolism is associated with abnormal protein turnover, consistent with previous research [40]. In the case of hypercholesterolemia, high levels of pyruvate and alanine were detected in PG rats compared to NG rats. These changes indicate that glycolysis and gluconeogenesis were inhibited and the energy consumption pattern was shifted to lipid oxidation in response to hypercholesterolemia [18,40,41].

The tricarboxylic acid (TCA) cycle is involved in linking the carbohydrate, fat, and protein metabolism. Further, the TCA cycle is the primary source that the body acquires energy and delivers raw materials for numerous biosynthesis activities in the body [42]. The aldol condensation of oxaloacetate in the TCA cycle will produce citrate, the end-product of an earlier turn of the cycle, and acetyl-CoA. Acetyl-CoA may be drawn from glucose via the glycolytic pathway, entering the mitochondria as pyruvate or from fatty acids that have undertaken β-oxidation. In the TCA cycle, citrate is transformed into isocitrate via cis-aconitate by aconitase. Then, isocitrate dehydrogenase (IDH) will convert isocitrate to α-ketoglutarate (αKG) in a decarboxylation reaction. The TCA cycle maintains the provision of a substantial source of cellular ATP and reduces equivalents that feed the electron transfer chain [43]. Citrate was found lower in PG rats as compared with NG rats as the intake of high cholesterol diet disrupts the normal energy metabolism. The decrease in citrate levels, which are the intermediate products of the tricarboxylic acid cycle, indicates that the TCA cycle is suppressed [44].

Acetate is the product of fatty acid oxidation. Significant elevation of acetate in PG rats, when compared with NG rats further, indicates enhanced fatty acid β-oxidation. Similar observations were also made in a study by Li et al. [45]. Enhanced fatty acid β-oxidation will upregulate the synthesis of ketone bodies. Ketone bodies (acetone, acetoacetate) were generated by the liver from fatty acid and then further converted into acetyl-CoA. In the next step, they enter the TCA cycle. The appearance of ketone bodies was considered to be one of the biomarkers of liver injury [46].

Choline is an essential nutrient for sustaining human health involving the mobilizing of fat from the liver. In animals, 95% of total tissue choline is used to form phosphatidylcholine (PC) via the Kennedy pathway. PC is essential for very-low-density lipoprotein (VLDL) packaging, exporting, and secreting triglyceride (TG) and acts as an intermediary to sustain a balance between plasma and liver fat [47]. Choline deficiency results in various disorders such as fatty liver and liver dysfunction, leading to elevations in liver aminotransferase serum concentrations. Additionally, insufficient supplies of choline can lead to the accumulation of TG in the liver and hepatic steatosis [48].

Moreover, choline is a neurotransmitter acetylcholine precursor and is essential in the structure of membrane phospholipids and lipoproteins. It performs essential functions in signal transduction, neurotransmitter synthesis, or lipid transport [42]. Additionally, plasma choline levels showed a positive correlation with serum TG and glucose levels, showing its involvement in multiple disease pathogenesis, including fatty liver, obesity, or cardiovascular disease [49]. Supplementation with 2% DDP showed an increased level of choline in DG rats compared to PG rats (*p* < 0.05). Hence, 2% of DDP might be of therapeutic potential to treat the elevated level of TG. This finding is in good agreement with a significant reduction of serum triglycerides seen in DG rats when compared with PG rats (*p* < 0.05) (Table 4). However, it also has several drawbacks, as DDP did not improve the metabolite levels of pyruvate, alanine, citrate, and acetate, metabolites which involve in energy metabolism and lipid metabolism.

## 5. Conclusions

This study demonstrated that although DDP was considered as waste, it still contains high dietary fibre and potent antioxidant properties related to the presence of phenolic compound (syringic acid, 4-hydroxybenzoic acid and gallic acid). The results of this study provide evidence that DDP ameliorates hypercholesterolemia by reducing total serum cholesterol, triglyceride, and LDL-C levels. DDP also has a good effect against oxidative stress by improving the antioxidant profile and lowers the inflammation after 30 days of treatment duration. The therapeutic ability of DDP-upregulated choline suggests that DDP acts via the choline metabolism in the metabolism dysfunction caused by hypercholesterolemia. In this study, ^1^H-NMR-based metabolomic analysis of urine of hypercholesterolemic rats uncovered the potential therapeutic effect of DDP. Future research is needed to investigate the mechanisms by which DDP impacts hypercholesterolemia disease progression. An improved understanding of the pathophysiology of hypercholesterolemia is vital to strengthen the strategy of DDP to combat hypercholesterolemia.

## Figures and Tables

**Table 1:** Treatment diets.

Ingredients (g)	Treatment Diet		
	ND ^1^	HC ^2^	HC + 2% DDP ^3^
Corn starch	180	170	170
Sucrose	500	500	500
Casein	120	120	120
Vitamin mixture	10	10	10
Mineral mixture	35	35	35
Cellulose	50	50	30
DL-methionine	3	3	3
Choline	2	2	2
Ghee	80	80	80
Corn oil	20	20	20
Cholesterol	-	10	10
Defatted dabai pulp	-	-	20
Total	1000	1000	1000
^4^ Energy (kcal/100g)	397	423	384

ND, normal diet; HC, high cholesterol diet; HC + 2% DDP, high cholesterol diet incorporated with 2% defatted dabai pulp.^1^ ND diet was given to normal rats group (NG), ^2^ HC diet was given to hypercholesterolemic positive control group (PG),^3^ HC + 2% DDP was given to hypercholesterolemic rats treated with 2% DDP group (DG). ^4^ Energy (kcal/100 g) represents the calories content in 100 g of diet.

**Table 2:** Selected phenolic compounds determined in the DDP extracts.

Phenolic Compound	Concentration (µg/mL)
Gallic acid	8.73 ± 0.13
4-hydroxybenzoic acid	61.46 ± 0.04
Syringic acid	89.87 ± 15.18

**Table 3:** Antioxidant profile in treatment diets.

Experimental Diet	ND	HC	2% DDP
FRAP (mM Fe/g extract)	7.369 ± 0.05 ^a^	7.200 ± 0.01 ^b^	11.197 ± 0.01 ^a,b^
TPC (mg GAE/g extract)	2.842 ± 0.12 ^a^	2.649 ± 0.05 ^b^	3.969 ± 0.01 ^a,b^
TFC (mg QE/g extract)	0.776 ± 0.00	0.698 ± 0.13	1.072 ± 0.00 ^a,b^

^a^ Indicates a statistically significant difference (*p* < 0.05) versus HC, ^b^ Indicate a statistically significant difference (*p* < 0.05) versus ND by Duncan’s multiple range tests. ND: Normal diet; HC: High cholesterol diet; 2% DDP: 2% defatted dabai pulp diet.

**Table 4:** The beneficial effects of DDP treatment diet on hypercholesterolemic rats.

Group	NG	PG	DG
TC (mmol/L)	1.57 ± 0.15 ^a^	2.12 ± 0.65 ^b^	1.37 ± 0.25 ^a^
TG (mmol/L)	1.97 ± 0.92	2.08 ± 0.65	1.18 ± 0.38 ^a^
LDL-C (mmol/L)	0.17 ± 0.06 ^a^	0.50 ± 0.19 ^b^	0.33 ± 0.11 ^a,b^
HDL-C (mmol/L)	1.36 ± 0.14	1.27 ± 0.53	1.25 ± 0.19
AST (U/L)	81.83 ± 4.17 ^a^	124.33 ± 23.90 ^b^	88.83 ± 13.73 ^a^
ALT (U/L)	20.33 ± 3.14 ^a^	30.33 ± 7.66 ^b^	22.33 ± 3.01 ^a^
TAS (U/mL)	2.24 ± 0.31 ^a^	1.95 ± 0.22 ^b^	2.51 ± 0.17 ^a^
SOD (ng/mL)	0.78 ± 0.13 ^a^	0.58 ± 0.02 ^b^	0.89 ± 0.12 ^a^
GPx (U/L)	295.98 ± 3.40 ^a^	282.72 ± 14.98 ^b^	301.23 ± 2.24 ^a^
CAT (U/mL)	12.96 ± 1.19 ^a^	4.26 ± 0.69 ^b^	20.13 ± 5.53 ^a,b^
CRP (ng/mL)	0.85 ± 0.16 ^a^	1.07 ± 0.20 ^b^	0.52 ± 0.05 ^a,b^
IL-6 (pg/mL)	315.32 ± 31.28 ^a^	364.97 ± 49.84 ^b^	251.11 ± 34.37 ^a,b^
α-TNF (pg/mL)	221.67± 14.36 ^a^	293.76 ± 20.41 ^b^	191.88 ± 6.49 ^a,b^

TC, total cholesterol; TG, triglyceride; NG, normal rats group; PG, hypercholesterolemic positive control group; DG, hypercholesterolemic rats treated with 2% DDP group. ^a^ Indicate a statistically significant difference (*p* < 0.05) versus PG group, ^b^ Indicate a statistically significant difference (*p* < 0.05) versus NG group by Duncan’s multiple range tests using SPSS for windows version 23. Results are given as Mean ± SD (*n* = 6).

**Table 5:** Potential marker in rat urine and their variations between groups.

Metabolites	VIP	PG/NG	DG/PG
Citrate	3.79	0.62 *	0.98
Acetate	2.14	1.47 **	1.07
Pyruvate	1.99	1.34 **	1.03
Choline	1.60	1.25 *	1.21 *
Cis-aconitate	1.38	1.20	0.98
Acetoacetate	1.27	1.22	1.02
Alanine	1.16	1.19 *	1.02
Lysine	1.09	1.22	0.98
Methylmalonate	0.91	1.13	1.01

NG, Normal rats group; PG, hypercholesterolemic positive control group; DG, hypercholesterolemic rats treated with 2% DDP. XXX/YYY means integral of metabolite in XXX group was divided by that of YYY group. The ratio over 1.00 indicated an increase, while ratio less than 1.00 indicated a decrease. Statistical analysis was performed by one-way ANOVA followed by the Duncan test. * *p* < 0.05; ** *p* < 0.01.

**Table 6:** Metabolic pathways.

Metabolic Pathways	*p*-Value	Impact Value
Citrate cycle (TCA cycle)	0.0002	0.19
Pyruvate metabolism	0.0085	0.27
Glycolysis/Gluconeogenesis	0.0118	0.13
Synthesis and degradation of ketone bodies	0.0327	0.60
Butanoate metabolism	0.0953	0.11

## Supplementary Materials

https://www.mdpi.com/2072-6643/12/11/3511/s1. Figure S1: HPLC chromatogram of DDP (a), DDP spiked with SA (b), DDP spiked with 4HBA (c) and DDP spiked with GA (d); Figure S2: Permutation Test (a–c), and misclassification probability test (d); Table S1: Assignment of ^1^H-NMR spectra peak of rat urine spectra
