# Supplementary material for: Effect of Defatted Dabai Pulp Extract in Urine Metabolomics of Hypercholesterolemic Rats
Source: Nutrients. 2020 Nov 14;12(11):3511. doi: 10.3390/nu12113511 (PMC7697915; doi:10.3390/nu12113511)
Supplement: Supplementary file 1 [file nutrients-12-03511-s001.zip › Supplementary Materials Nutrients/Table S1.docx]

**Table S1.** Assignment of ^1^H-NMR spectra peak of rat urine spectra

| **Metabolites** | **δ^1^H (ppm) and multiplicity** |
| --- | --- |
| Leucine | 0.94 (t) |
| 3-hydroxybutyrate | 1.18 (d) |
| Methylmalonate | 1.22 (d) |
| Threonine | 1.3 (d) |
| Lactate | 1.34 (d) |
| Lysine | 1.42 (m) |
| Alanine | 1.46 (d) |
| Acetate | 1.9 (s) |
| Acetone | 2.18 (s) |
| Acetoacetate | 2.26 (s) |
| Pyruvate | 2.34 (s) |
| Succinate | 2.38 (s) |
| Citrate | 2.66 (d), 2.5 (d) |
| Dimethylamine | 2.7 (s) |
| N, N-Dimethylglycine | 2.9 (s) |
| 2-oxoglutarate | 2.98 (t), 2.42 (t) |
| Creatine | 3.02 (s) |
| cis-Aconitate | 3.1 (s) |
| Choline | 3.18 (t) |
| Trimethylamine N-oxide | 3.22 (s) |
| Taurine | 3.42 (t) |
| Glucose | 3.82 (m) |
| Creatinine | 4.02 (s) |
| Trigonelline | 4.42 (s) |
| 1-Methylnicotinamide | 4.46 (s) |
| Allantoin | 5.38 (s) |
| 3-Indoxylsulfate | 7.26 (t) |
| N-Phenylacetylglycine | 7.34 (m) |
| Hippurate | 7.62 (t), 7.54 (t) |

s: singlet, d: doublet. t: triplet, m: multiplet,
