# Supplementary figures and images for: Effect of Defatted Dabai Pulp Extract in Urine Metabolomics of Hypercholesterolemic Rats
Source: Nutrients. 2020 Nov 14;12(11):3511. doi: 10.3390/nu12113511 (PMC7697915; doi:10.3390/nu12113511)

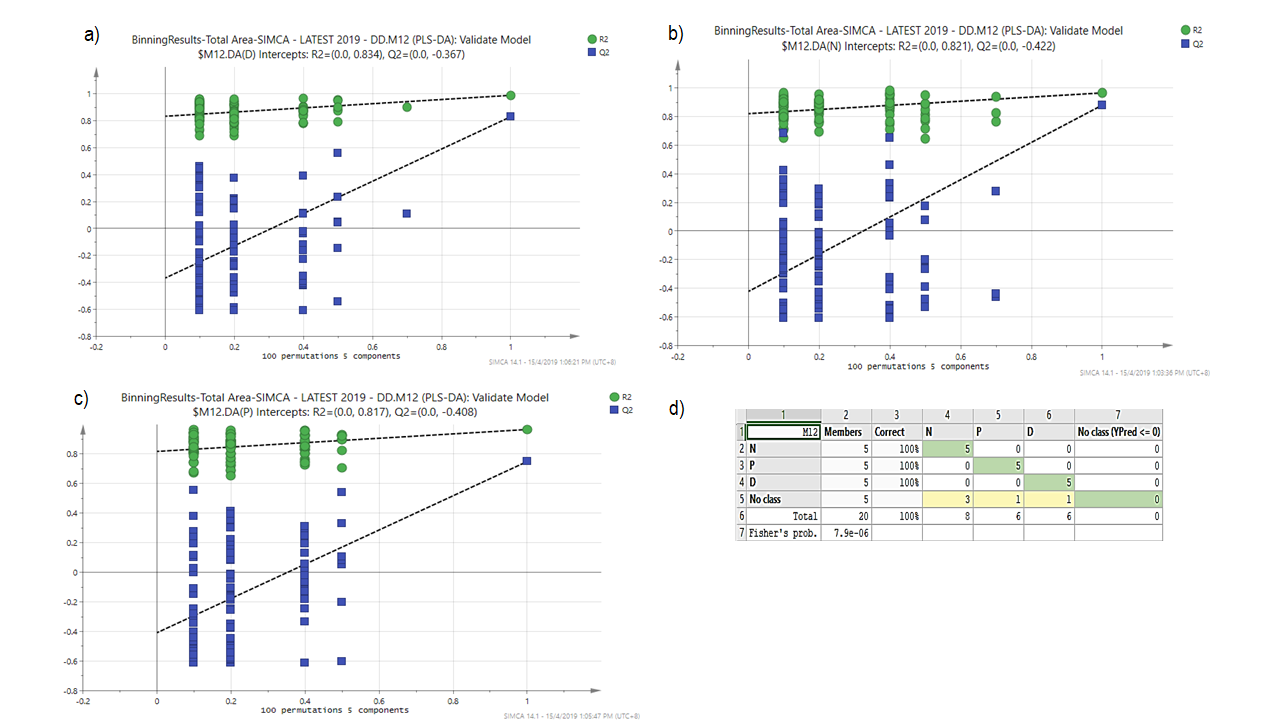

Supplement: Supplementary file 1 [file nutrients-12-03511-s001.zip › Supplementary Materials Nutrients/Figure S1.tif]

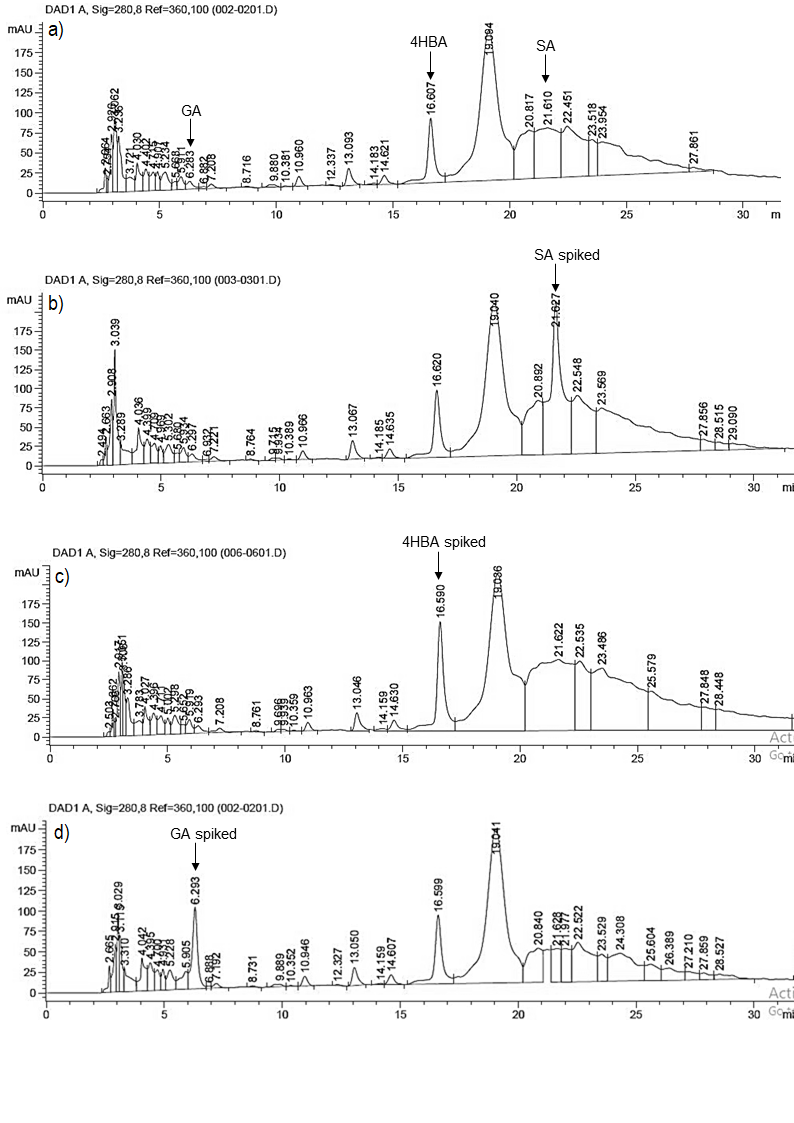

Supplement: Supplementary file 1 [file nutrients-12-03511-s001.zip › Supplementary Materials Nutrients/Figure S2.tif]
